# Supplementary material for: Developing Feasible, Locally Appropriate Socioeconomic Support for TB-Affected Households in Nepal
Source: Trop Med Infect Dis. 2020 Jun 10;5(2):98. doi: 10.3390/tropicalmed5020098 (PMC7345977; doi:10.3390/tropicalmed5020098)
Supplement: Supplementary file 1 [file tropicalmed-05-00098-s001.pdf]

Agenda of the workshop

BNMT Nepal, 11th-12th September 2019

Workshop to develop a shortlist of potential socioeconomic interventions for TB-affected households in Nepal

A G E N D A

Day 1

Time	Activities
9:00-9:15	Welcome, objective sharing, and opening remarks
9:45-10:15	National Social Protection Policy/ Program
10:15-10:45	Findings on catastrophic costs in Nepal
11:00 – 11:30	Wellcome Trust research designing TB socioeconomic support in Nepal
11:30-12:00	Social Protection activities in TB in Nepal
12:00-12:30	Open Discussion
13:30 – 14:00	TB Scenario in Nepal
14:00 - 14:30	Overview of Group work to "Develop locally-appropriate socioeconomic interventions to support TB-affected households in Nepal"
14:30-15:45	Group Discussion on developing locally-appropriate socioeconomic interventions to support TB-affected households in Nepal
15:45 – 16:00	**T E A B R E A K**
16:00 – 16:45	Group feedback on developing locally-appropriate socioeconomic interventions to support TB-affected households in Nepal
16:45 – 17:00	Wrap-up of Day 1
6 pm onwards	Snack and refreshments

Day 2

Time	Activities
9:00 – 9:15	Recap of Day 1

9:15 – 9:45	Synthesis of expert group findings
9:45 – 11:00	Group discussions on the findings
11:30-12:15	Group recommendations on creating a shortlist of potential socioeconomic interventions for TB-affected households in Nepal
12:15-12:45	Open Discussion
12:45-13:00	Summary of key points of the workshop and next steps
13:00 –13:15	Closing remarks

Workshop group-work materials and instructions

DEVELOPING A SHORTLIST OF POTENTIAL SOCIOECONOMIC INTERVENTIONS FOR TB-AFFECTED HOUSEHOLDS IN NEPAL

Instructions for Group Work on Afternoon of First Day

Groups 1 and 2

Group Work Topic: What would be the best intervention/s to address the psychosocial impact of TB in Nepal?

Background: As has been demonstrated in other countries around the world, today's presentations and Wellcome Trust Seed Award findings have suggested that people with TB in Nepal are commonly affected by stigma (including enacted stigma from others or self-stigmatisation), depression, and anxiety.

Questions to answer in the group:

1. Are there any existing interventions (related to TB or any other illnesses) to address the psychosocial impact of TB? If so, how could these be improved in the future?
2. What would be the best intervention/s in the Nepal context to reduce the psychological and social consequences of TB?
3. By whom would the intervention/s be funded and how would the intervention be delivered (e.g. who would implement and at what time points during TB illness and treatment)?

With respect to the above questions, consider in your responses the *acceptability* of the intervention to people with TB and the *feasibility/sustainability* of the intervention for those delivering it (e.g. budget / person-power required / likelihood of scale-up)

Presentation of group findings: a member of BNMT staff will be on hand to take notes relating to the group work discussion and make 4 powerpoint slides in answer to the above questions. Other BNMT staff and workshop leads will be available if you need any other clarifications. **The group should nominate a member to feedback the slides to the workshop participants at the end of the session**. There are 75 minutes to complete the group work.

Groups 3 and 4

Group Work Topic: What would be the best intervention/s to address the economic impact of TB in Nepal?

Background: As has been demonstrated in other countries around the world, today's presentations and Wellcome Trust Seed Award findings have suggested that people with TB in Nepal have a high prevalence of catastrophic costs relating to seeking diagnosis and care, including out-of-pocket expense but also lost income.

Questions to answer in the group:

1. What are the existing interventions to address the economic impact of TB in Nepal? How could these be adapted/improved?
2. What would be the best intervention/s in the Nepal context to reduce the economic consequences of TB?
3. By whom would the intervention/s be funded and how would they be delivered (e.g. who would implement and at what time points during TB illness and treatment)?

Consider in your responses the *acceptability* of the intervention to people affected by TB and the *feasibility/sustainability* of the intervention for those delivering it (e.g. budget / person-power required / likelihood of scale-up).

Presentation of group findings: a member of BNMT staff will be on hand to take notes relating to the group work discussion and make 4 powerpoint slides in answer to the above questions. Other BNMT staff and workshop leads will be available if you need any other clarifications. **The group should nominate a member to feedback the slides to the workshop participants at the end of the session**. There are 75 minutes to complete the group work.

Groups 5 and 6

Group Work Topic: Who would be the recipients of a socioeconomic intervention for people with TB?

Background: Currently, Nepal has existing support packages in place for people with MDR-TB, including travel vouchers, accommodation and nutritional support. The Seed Award research suggests that people with drug-sensitive TB are also vulnerable to the socioeconomic impact of TB. Given budgets for socioeconomic support are limited, it is worth considering if intervention/s should be targeted to certain high-risk groups or available to all people with TB.

Questions to answer in the group:

1. Apart from people with MDR-TB, which other people with TB do you think are most vulnerable to socioeconomic impact?
2. Would you recommend socioeconomic support to all people with TB or only certain groups that you have identified above?
3. What are the benefits and drawbacks of a socioeconomic intervention that has a “floor” (basic package) for all people with TB plus an enhanced package for only those at high-risk?

With respect to the above questions, consider in your responses the *feasibility, sustainability, and logistics* of delivering the intervention (e.g. budget / paperwork and bureaucracy / complexity / person-power required / likelihood of scale-up)

Presentation of group findings: a member of BNMT staff will be on hand to take notes relating to the group work discussion and make 4 powerpoint slides in answer to the above questions. Other BNMT staff and workshop leads will be available if you need any other clarifications. **The group should nominate a member to feedback the slides to the workshop participants at the end of the session**. There are 75 minutes to complete the group work.
